# Three‐Year Outcomes After Temperature‐Controlled Radiofrequency Treatment of Nasal Airway Obstruction

**DOI:** 10.1002/oto2.70111

**Published:** 2025-04-07

**Authors:** William C. Yao, Randall Ow, Michael J. Sillers, Nathan E. Nachlas, Curtis D. Johnson, Dale Ehmer, Jordan Pritikin, Henry P. Barham

**Affiliations:** ^1^ Department of Otorhinolaryngology–Head and Neck Surgery McGovern Medical School at the University of Texas Health Science Center Houston Texas USA; ^2^ Sacramento Ear, Nose and Throat Medical and Surgical Group Roseville California USA; ^3^ Alabama Nasal and Sinus Center Birmingham Alabama USA; ^4^ ENT and Allergy Associates of Florida Boca Raton Florida USA; ^5^ ENT and Allergy Associates of Florida Plantation Florida USA; ^6^ Ear, Nose, and Throat Associates of Texas McKinney Texas USA; ^7^ Chicago Nasal and Sinus Center Chicago Illinois USA; ^8^ Sinus and Nasal Specialists of Louisiana Baton Rouge Louisiana USA

**Keywords:** nasal airway obstruction, nasal valve, nasal valve collapse, radiofrequency, rhinoplasty, septoplasty

## Abstract

**Objective:**

To evaluate the long‐term safety and effectiveness of temperature‐controlled radiofrequency (TCRF) treatment of nasal valve collapse (NVC) in patients with nasal airway obstruction (NAO).

**Study Design:**

This is an extended follow‐up from a prospective, multicenter, single‐arm study. The initial study included participants from 12 sites across the United States who were followed for 24 months and additionally agreed to participate in the extended 36‐month follow‐up.

**Setting:**

Procedure was performed in‐office with an in‐person follow‐up at 3 months and subsequent follow‐up assessment remotely.

**Methods:**

Participants received TCRF treatment of only the nasal valve and participated in the extended 36‐month follow‐up. The effect of TCRF treatment was determined by analyzing changes in nasal obstruction symptom evaluation (NOSE) score at each follow‐up compared to the baseline.

**Results:**

Of the 122 participants in the primary study, 66 participated in the extended 36‐month follow‐up. Compared to baseline, there was a 52.6% decrease in the NOSE score at 36 months (mean change −45.3 [95% CI −52.3 to −38.3]; *P* < .001), and 83.3% of the participants met the criteria for treatment response at 36 months, as defined by the study endpoint. Post hoc sensitivity analysis of the treatment response for all participants from the time of enrollment was 73.9%. No device or procedure‐related adverse events or serious adverse events were reported in the interval between 24 and 36 months.

**Conclusion:**

TCRF treatment of only the nasal valve resulted in significant and durable improvement in NAO symptoms through 36 months in participants with NAO due to NVC.

Nasal airway obstruction (NAO) is a common condition with a negative impact on quality of life.^1^ NAO can be caused by different nasal structural disorders such as septal deviation, turbinate enlargement, septal swell body, and nasal vestibular stenosis; however, nasal valve collapse (NVC) plays one of the most significant roles in many patients.^2^ NVC is primarily caused by the weakness of the cartilage surrounding the nasal valve (NV). The NV area is the narrowest part of the nose; hence, according to Poiseuille's law, a slight constriction in this area can result in an exponential increase in airflow restriction within the nasal airway. Therefore, small increases in the NV area can result in a significant improvement in the symptoms of NAO and the quality of life of patients.^3^, ^4^

Common medical management of NVC includes the use of external nasal dilating strips or internal nasal dilators, which require continuous usage and frequent replacement. Surgical treatments include cartilage grafting, suspension techniques, and patency‐maintaining implants, which are invasive and prone to risks such as bleeding, implant/graft extrusion, infection, cosmetic changes, persistent discomfort, and increased procedural costs.

Temperature‐controlled radiofrequency (TCRF) is a minimally invasive treatment for NAO that tightens the upper lateral cartilage at the lateral nasal wall, resulting in an increase in the NV area. A previous study showed that TCRF treatment of the NV using the VivAer system (Aerin Medical, Inc.) resulted in a significantly greater reduction in NAO symptoms compared to sham control through 12 months postprocedure.^5^, ^6^ Additionally, in an earlier publication of this trial, TCRF treatment of the NV led to significant improvement in NAO symptoms observed at 3 months that was maintained through 24 months.^7^ Here, we present long‐term outcomes for TCRF treatment of NVC in a cohort of patients with NAO through 36 months.

## Methods

### Study Design and Participants

Participants in this extended follow‐up study were invited to participate after completing the 24‐month follow‐up in the initial study. This single‐arm prospective study was conducted at 12 locations across the United States, approved by the Western Institutional Review Board (ID: 20192967), and registered on clinicaltrials.gov (NCT04277507). Site investigators were board‐certified otolaryngologists, and participants were recruited from their respective clinical practices. All participants were diagnosed with severe or extreme NAO primarily attributed to NVC at the time of enrollment. Complete inclusion and exclusion criteria have been previously reported^6^ and are presented in Supplemental Table S1, available online. Participants who completed the primary study but did not enroll for the 36‐month follow‐up are referred to as “nonparticipants,” whereas participants enrolled through the 36 months are referred to as “participants.”

### Study Procedure

**Figure 1:**
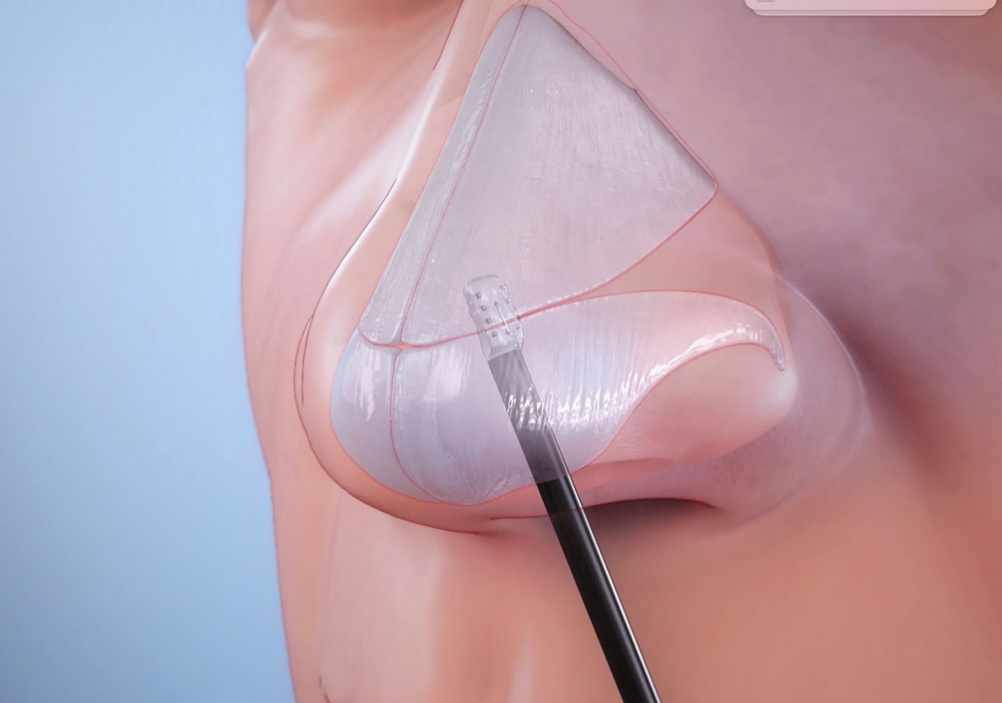
Temperature‐controlled radiofrequency device positioned at the lateral nasal valve treatment area.

The minimally invasive TCRF procedure was performed as a single treatment in an office setting using the VivAer system as previously described.^5^, ^6^, ^8^ In summary, the device delivers radiofrequency energy to the target tissue through bipolar electrodes, controlling for a maximum temperature of 60°C. Prior to the procedure, local anesthesia (eg, 1% lidocaine with epinephrine) was applied according to the preference of the investigator. The stylus was placed on the lateral wall, and treatment was administered at nonoverlapping areas of the lateral NV area with standardized treatment settings of 4 W power, 18‐second treatment time, and 12‐second cooling time (Figure 1). Participants received an average of approximately four treatments per nostril. Although the procedure may be performed with nasal endoscopy or anterior rhinoscopy, the study protocol required it to be performed endoscopically for image collection.

### Outcome Assessments

Results, demographics, and key participant characteristics were analyzed for all eligible participants enrolled in the study, including those participating participants and nonparticipants.

NAO symptoms were measured using the nasal obstruction symptom evaluation (NOSE) score, a validated patient‐reported outcome measure. The severity categories of the NOSE scale indicate mild (range, 5‐25), moderate (range, 30‐50), severe (range, 55‐75), or extreme (range, 80‐100) nasal obstruction.^9^ The reduction of NAO symptoms was defined as a decrease (ie, improvement) in the NOSE scale score at each follow‐up timepoint compared to the baseline score. Responders were defined as participants with ≥20% decrease in the NOSE scale score or an improvement in ≥1 severity category from baseline. The percentage of participants with a positive shift in symptom severity category compared to baseline was calculated. As a pragmatic study, the use of medications was not controlled. However, medications taken for nasal obstruction symptoms were recorded at baseline and compared with those taken at the 36‐month follow‐up timepoint. Medications were grouped into seven categories (antihistamines, decongestants, leukotriene inhibitors, steroid nasal sprays, anticholinergic nasal sprays, allergy immunotherapy, and other). Adverse events (AEs) were recorded throughout the study (Supplemental Table S2, available online).

### Statistical Analysis

Demographic data and NOSE scores for participants and nonparticipants were analyzed at baseline and 36 months. Statistical analysis was performed with SAS 9.4 (SAS Institute). Continuous variables are reported using a descriptive analysis of mean and standard deviations. Frequencies and percentages are reported for categorical variables. A linear mixed model repeated measures (MMRM) analysis with multiple comparisons (Dunnett‐Hsu) of timepoints was used to evaluate the NOSE score at each follow‐up compared to the baseline score. Adjusted (least square) mean and 95% confidence intervals (CI) of the NOSE score from the MMRM analysis were reported at baseline and at each follow‐up. Mean change and % change from baseline were calculated and reported for NOSE scores. The difference in NOSE score between participants and nonparticipants at baseline and at the final follow‐up was assessed using the Wilcoxon rank‐sum test. Generalized estimating equations (GEEs) were used to assess repeated multinomial ordered category distributions of the NOSE scale severity class. To assess the differences between severity categories between follow‐up visits, an exploratory analysis was performed using the odds ratios derived from the GEE. Significance was accepted at .05 alpha.

## Results

### Participants

**Figure 2:**
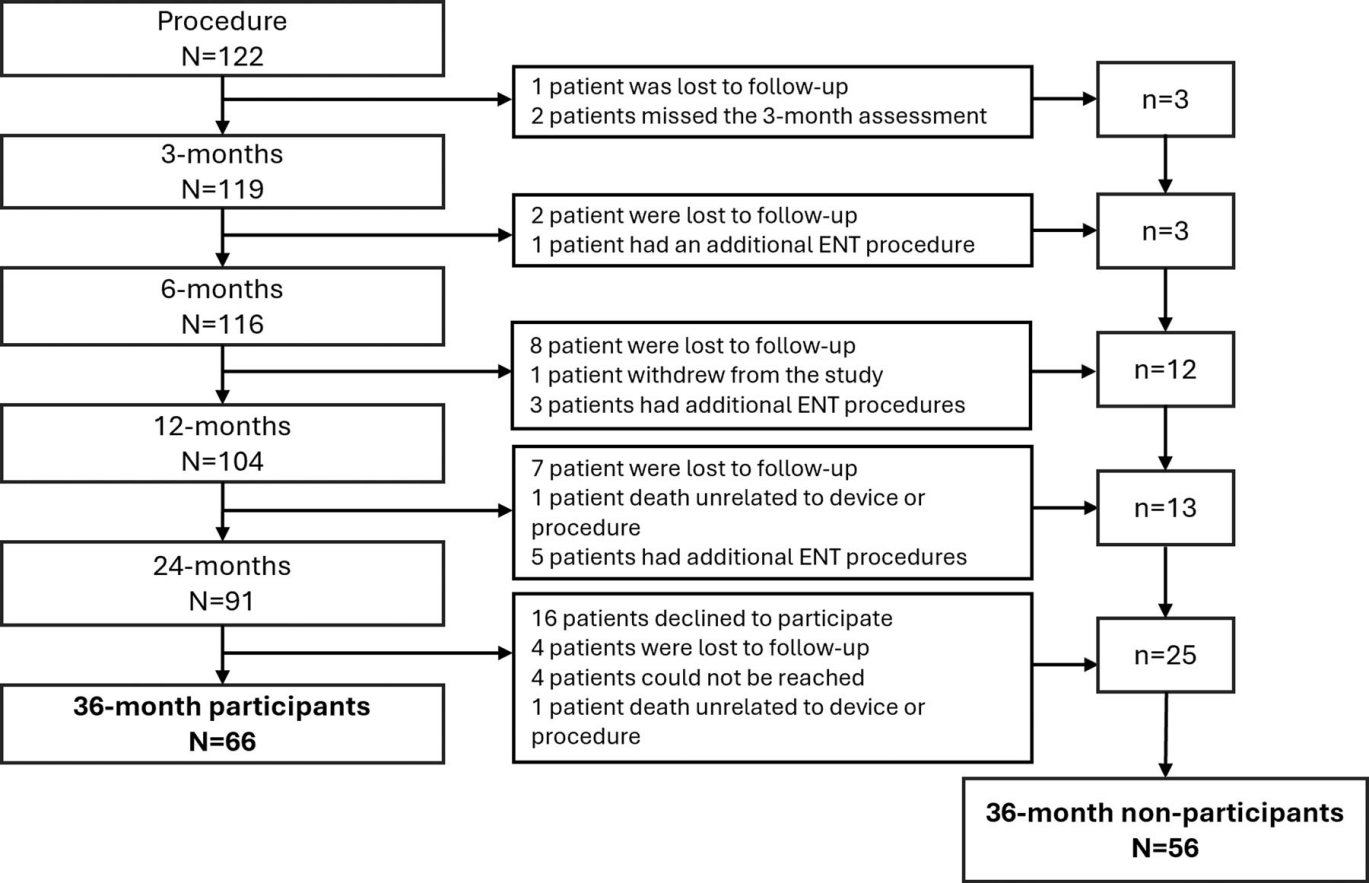
Participant disposition through 36 months. “Lost to follow‐up” participants completed the 24‐month follow‐up study and agreed to participate in the 36‐month follow‐up study but did not respond to requests to complete the questionnaires. The “could not be reached” participants completed the 24‐month follow‐up but did not respond to requests to participate in the 36‐month follow‐up study.

Of the 122 participants enrolled in the primary study, 91 participated through 24 months, and 66 participated in the extended 36‐month follow‐up study (Figure 2). All participants who declined to participate in the 36‐month follow‐up had completed the study and exited as originally planned through the 24‐month follow‐up per the protocol. The reasons specified for those who did not reconsent to the 36‐month follow‐up study are listed in Supplemental Table S3, available online. Demographic information, treatment history, and characteristics, including nasal exam findings, are presented for the participants and nonparticipant groups (Table 1). There was no significant difference between participants and nonparticipants except for the baseline nasal exam of turbinate enlargement (*P* = .04) and nasal swell body (NSB) enlargement (*P* = .05), which was more common in the participants. Additional ear, nose, and throat(ENT) procedures are listed in Supplemental Table S4, available online.

**Table 1:** Participant Demographics and Characteristics

Characteristics	Participants (N = 66)	Nonparticipants (N = 56)	*P*‐value
Age, y	49.8 (17.2)	50.5 (15.7)	.81
BMI, cm/m^2^	28.1 (7.4)	26.9 (5.7)	.35
Sex, n (%)			.86
Female	34 (51.5)	30 (53.6)	
Male	32 (48.5)	26 (46.4)	
Race, n (%)			.79
White	59 (89.4)	48 (85.7)	
Asian	1 (1.5)	2 (3.6)	
Asian, white	1 (1.5)	1 (1.8)	
Black/African American	1 (1.5)	3 (5.4)	
Black/African American, white	1 (1.5)	‐	
Declined to answer	3 (4.5)	2 (3.6)	
Prior nasal procedures,^a^ n (%)			
Turbinate reduction	14 (21.2)	14 (25)	.67
Polyp removal	5 (7.6)	1 (1.8)	.22
Septoplasty	25 (37.9)	19 (33.9)	.71
Rhinoplasty	6 (9.09)	3 (10.7)	.51
Sinuplasty	6 (9.1)	6 (10.7)	.77
Baseline nasal exam, n (%)^b^			
Nasal valve collapse	65 (100)	56 (100)	‐
Septal deviation	22 (33.8)	13 (23.2)	.23
Nasal valve stenosis	38 (58.5)	27 (48.2)	.28
Turbinate enlargement	22 (33.8)	9 (16.1)	.04
Septal turbinate (NSB)	25 (38.5)	12 (21.4)	.05
Nasal polyps	2 (3.1)	‐	.50
NOSE score, mean (SD)			
Baseline NOSE score	80.4 (12.2)	80.3 (12.7)	.96
NOSE score at last visit	35.1 (29.6)	30.0 (27.2)	.51

Abbreviations: BMI, body mass index; NOSE, nasal obstruction symptom evaluation; NSB, nasal swell body.

^a^ Continuous variables are presented as mean and standard deviation (SD); categorical measures are presented as numbers (% of total).

^b^ Numbers and percentages represent participants providing a “yes” response; one individual in the participant group did not have a baseline nasal examination.

### Outcome Assessments

#### NOSE Scale Score

**Figure 3:**
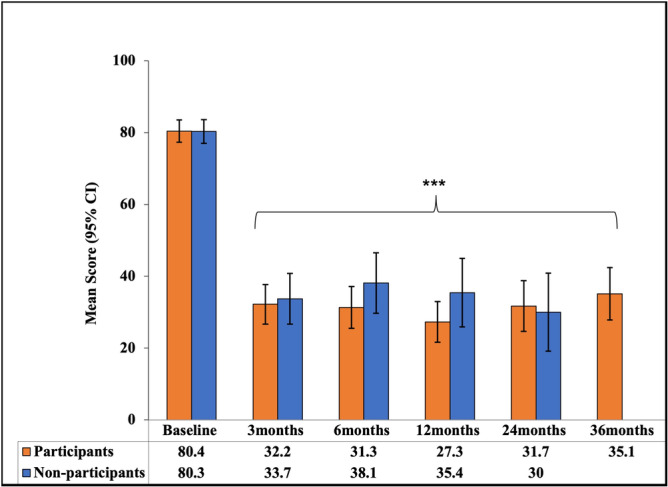
Participants' adjusted (least squared) mean scores for the nasal obstruction symptom evaluation (NOSE) scale at baseline (n = 66), 3 months (n = 65), 6 months (n = 66), 12 months (n = 66), 24 months (n = 66), and 36 months (n = 66) and nonparticipants’ least squared mean scores for the NOSE scale at baseline (n = 56), 3 months (n = 54), 6 months (n = 50), 12 months (n = 36), and 24 months (n = 24), following temperature‐controlled radiofrequency treatment. *A significant difference from the baseline score in participants (*P* < .001). Bars represent 95% CI.

**Figure 4:**
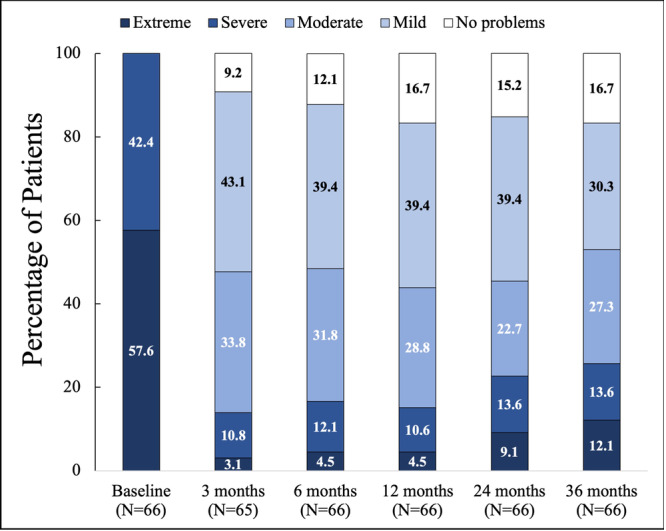
Percentage of participants for each nasal obstruction symptom evaluation severity category at baseline, 3, 6, 12, 24, and 36 months following temperature‐controlled radiofrequency treatment of nasal valve collapse. No significant changes in symptom severity distributions were seen between successive study visits, and no significant changes were seen in symptom severity distributions, with the exception of the comparison of the 12‐month and 36‐month visits (*P* = .029) and all follow‐up visits compared to baseline (*P* < .001).

**Figure 5:**
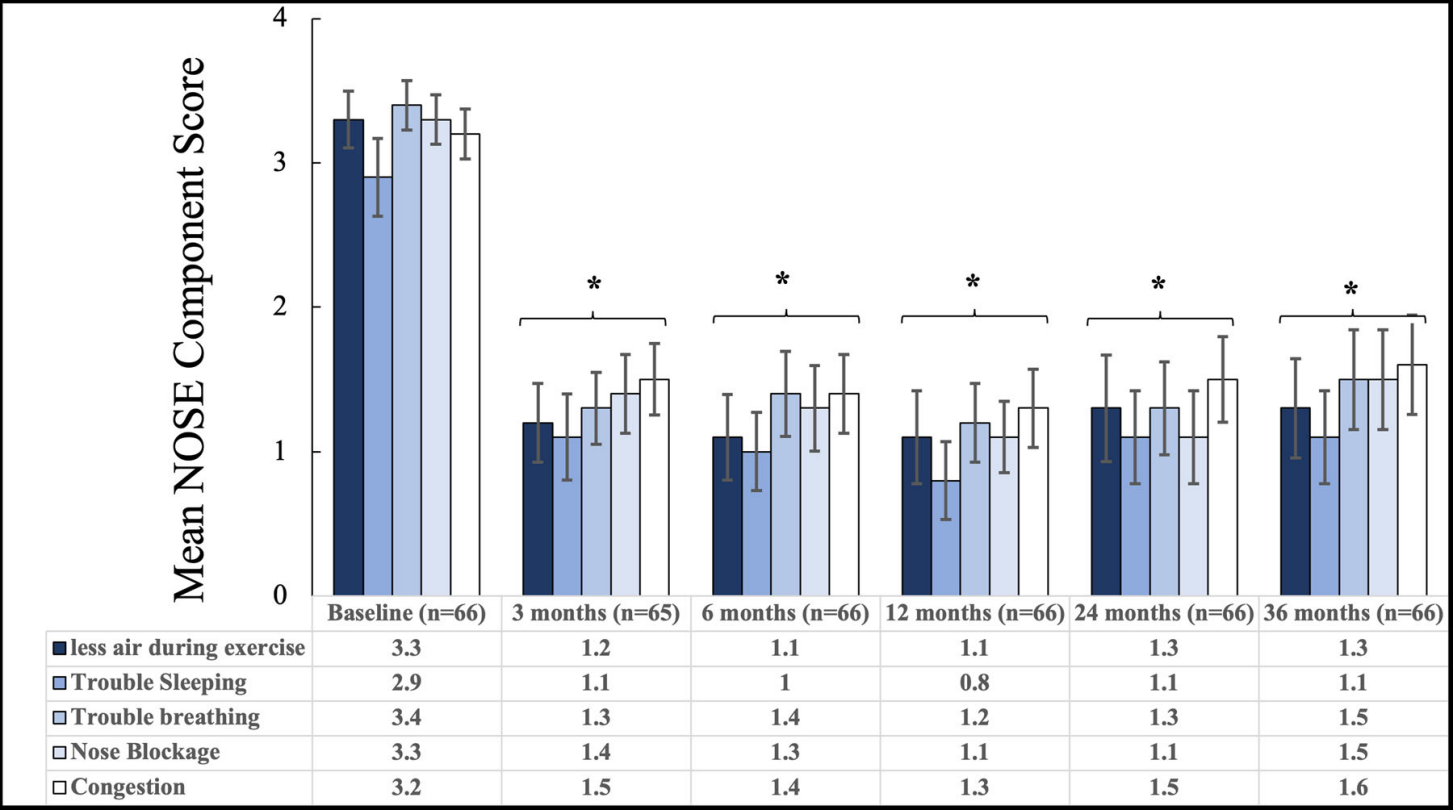
Participants’ mean score for each nasal obstruction symptom evaluation (NOSE) score component at baseline, 3, 6, 12, 24, and 36 months following temperature‐controlled radiofrequency treatment of the nasal valve. Bars represent 95% CI. *A significant difference at all timepoints compared to the baseline score (*P* < .001).

Participants in the follow‐up study had a significant reduction in NOSE score from a baseline mean score of 80.4 to an adjusted mean score of 35.1 (95% CI, 27.8‐42.3; *P* < .001) at 36 months. This represents a mean change of −45.3 (95% CI, −54.2 to −36.4; *P* < .001), a 52.6% improvement from baseline. This decrease in NOSE score is consistent with those observed in participants at 3 months (59.7% improvement, mean change −48.5 [95% CI, −55.8 to −40.7]), 6 months (61.4% improvement, mean change −49.1 [95% CI, −56.5 to −41.6]), 12 months (66.4%, mean change −53.0 [95% CI, −60.2 to 45.9]), and 24 months (61.6% improvement, mean change −48.7 [95% CI, 57.1‐40.3]) (Figure 3). At baseline, 42.4% and 57.6% of participants had severe and extreme NAO symptoms, respectively. At 36 months, the severity categories of the NOSE of the participants were the following: 16.7% (no problem), 30.3% (mild), 27.3% (moderate), 13.6% (severe), and 12.1% (extreme). Figure 4 shows the percentage of participants in each severity category at 3 through 36 months. Although there were numeric differences in the distributions of symptom severity categories over time, exploratory analysis of pairwise comparisons of the differences between successive study visits showed that the differences were not statistically significant. When comparing the distributions of each timepoint to all other timepoints, only the comparison of the 12‐month and 36‐month visits reached statistical significance (*P* = .029). All comparisons for distributions at each timepoint compared to baseline were statistically significant (*P* < .0001) (Supplemental Table S5, available online). Additionally, 95.4%, 90.9%, 92.4%, 89.4%, and 83.3% of the participants were responders to treatment at 3, 6, 12, 24, and 36 months, respectively. Additionally, a significant decrease was observed in all NOSE score components at each time timepoint compared to baseline, *P* < .001 (Figure 5). To account for the potential bias in the responder group, an exploratory sensitivity analysis was conducted. In this analysis, subjects who underwent additional ENT procedures at any time during the study or reported severe or extreme NOSE scores (and had 36‐month data) were imputed as “nonresponders.” Despite imputing these patients as nonresponders, 65.3% of the patients were still responders to treatment at 36 months postprocedure (Supplemental Table S6, available online). When we examine the nonresponse rate of these subjects in the overall population (all participants [N = 119] enrolled through 36 months), we obtain a response rate of 73.9% for the study and 26% nonresponse rate.

Nonparticipants had a 61.3% decrease in NOSE score with a mean change of −46.9 (SD 26.5) at their final follow‐up visit at 24 months after the procedure, with 91.7% classified as responders to treatment. There was no significant difference in NOSE scale scores between participants at 36 months and nonparticipants at their final follow‐up of 24 months; *P* = .51 (Table 1).

#### Safety

The standardized definition (ISO14155) of AEs was used to document and classify events throughout this study. AEs with any potential relationship to the device and/or procedure are reported; no new device‐ or procedure‐related AEs were reported in the interval between 24 and 36 months as evaluated by patient self‐assessments, and no serious AEs were reported at any time throughout the study. (See Supplemental Table S2, available online, for previously reported AEs.)

#### Medication Use

Eight participants started on a new medication after the baseline visit and remained on it at the time of the 36‐month follow‐up visit. A total of 3 (4.5%) started on an antihistamine, 3 (4.5%) started on a steroid nasal spray, 1 (1.5%) started on an anticholinergic, and 1 (1.5%) started on an oral combination medication: two started the new medication at 3 months postprocedure, one started at 6 months, two started at 12 months, two started at 24 months, and one started after 24 months follow‐up. Of the eight patients starting a new medication after TCRF treatment, all had comorbid allergic rhinitis/seasonal allergies at baseline or indicated that the medication started was to treat allergic rhinitis, postnasal drip, or allergies which may contribute to NAO but has different treatment implications in relation to NAO caused by NVC. Supplemental Table S7, available online, provides additional detail for these patients through the 36‐month follow‐up period.

Compared to baseline, at 36 months, the majority of participants had either stopped or reduced their dose of leukotriene inhibitors, decongestants, and steroid nasal sprays, which were reported at different timepoints throughout the study (Table 2). Specifically, 34.6% of participants had either stopped or reduced their dose of antihistamine, 50% of participants had either stopped or reduced their dose of decongestant, 58.3% of participants had either stopped or reduced their dose of leukotriene inhibitors, 57.7% of participants had either stopped or reduced their dose of nasal steroid nasal sprays, and 66.6% of participants had stopped their dose of “other” medication.

**Table 2:** Participant Medication Usage

Medication class	Used at baseline N (%) (N = 66)	Medication started after baseline N (%)^a^	Medication change category^b^	No. of participants N (%)^c^
Antihistamines	26 (39.4)	3 (4.5)		
			No change in medication	14 (53.8)
			Increased dose	0 (0.0)
			Decreased dose	5 (19.2)
			Stopped medication	4 (15.4)
Decongestants	4 (6.1)	0 (0.0)		
			No change in medication	2 (50.0)
			Increased dose	0 (0.0)
			Decreased dose	1 (25.5)
			Stopped medication	1 (25.5)
Leukotriene inhibitors	12 (18.2)	0 (0.0)		
			No change in medication	5 (41.7)
			Increased dose	0 (0.0)
			Decreased dose	1 (8.3)
			Stopped medication	6 (50.0)
Nasal steroid sprays^d^	26 (39.4)	3 (4.5)		
			No change in medication	11 (42.3)
			Increased dose	0 (0.0)
			Decreased dose	7 (26.9)
			Stopped medication	8 (30.8)
Nasal anticholinergic sprays	2 (3.0)	1 (1.5)		
			No change in medication	2 (100.0)
			Increased dose	0 (0.0)
			Decreased dose	0 (0.0)
			Stopped medication	0 (0.0)
Immunotherapy	1 (1.5)	0 (0.0)		
			No change in medication	1 (100.0)
			Increased dose	0 (0.0)
			Decreased dose	0 (0.0)
			Stopped medication	0 (0.0)
Other^e^	3 (4.5)	1 (1.5)		
			No change in medication	1 (33.3)
			Increased dose	0 (0.0)
			Decreased dose	0 (0.05)
			Stopped medication	2 (66.6)

^a^ Percentage was calculated by using the total number of participants (N = 66) as the denominator reflecting what the participants were taking at the 36‐month follow‐up visit.

^b^ Increased medication is defined as an increase in either the dosage or frequency of a medication compared to what was taken at the baseline evaluation.

^c^ Percentage was calculated by using the number of participants who reported using each medication at baseline as the denominator.

^d^ Steroid sprays include intranasal compound sprays.

^e^ Other equals the sum of “antihistamine/decongestant,” expectorant, and other combination medications.

## Discussion

This long‐term follow‐up study demonstrates a durable and consistent improvement in NAO symptoms, as demonstrated by significant reductions in NOSE scale scores among participants with NVC through 36 months after TCRF treatment limited to the NV. There was also a significant decrease in the severity of NAO symptoms among participants at 36 months. This improvement in NAO symptoms is comparable to previous studies using TCRF and with NOSE score changes reported following functional rhinoplasty of the NV (ranging from −54.8 to −42.3).^5^, ^6^, ^7^, ^8^, ^10^, ^11^ In a meta‐analysis, Han et al identified that participants undergoing TCRF and functional rhinoplasty surgery focused on the NV had similar treatment improvements in NOSE scores at 12 months with a weighted mean difference of −48.8 and −45.3 at 12 months, respectively.^12^ When examining results, participants in this present study reported a comparable significant mean change of −45.3 in NOSE scores 36 months after the procedure, demonstrating excellent durability of the treatment effect. The minimally invasive nature of the TCRF procedure for the repair of the NV makes it possible to perform in either a surgical environment or an office setting and can be done via nasal endoscopy or anterior rhinoscopy, according to physician preference.

At baseline, all participants were categorized as having “severe” or “extreme” NAO symptoms and 36 months postprocedure; 74.3% of the participants had their NAO symptoms categorized as “no problem” to “moderate.” Although we acknowledge that numerically, the percentage of participants in the mild symptom severity category decreased over time and the extreme category increased over time, a post hoc exploratory pairwise comparison analysis did not show significant changes in these distributions between successive study visits, and no significant changes were seen in symptom severity category distributions, with the exception of 12‐month versus 36‐month visit (*P* = .029). Despite these findings, most participants continued to show benefits through 36 months posttreatment.

Participants also reported a notable reduction in medication usage at 36 months postprocedure.

Our analysis also shows that 91.7% of the nonparticipants responded to the TCRF procedure with a significant reduction in NOSE scores. Comparing the two groups, there was no significant difference in mean NOSE scores between participants and nonparticipants at baseline or at their final follow‐up.

To better understand these results, it is necessary to consider additional factors that may have influenced NAO outcomes, including subsequent procedural interventions and medication use. A total of 14 enrolled patients were identified as having nasal procedures postenrollment through the 24‐month follow‐up period. None of the participants in the extended 36‐month follow‐up reported undergoing additional nasal procedures between the 24‐month and 36‐month visits. Of the 14 patients with postenrollment nasal procedures, 9 participants in the 36‐month follow‐up had supplemental ENT procedures, including 3 patients having interventions targeting the NV and the remaining patients received procedures such as inferior turbinate reduction, sinus surgery, posterior nasal nerve ablation, septal swell body ablation, and septoplasty to address other contributing factors to NAO. Of the remaining five patients not participating in the 36‐month follow‐up, two had procedures targeting the NV, see Supplemental Table S4, available online.

The fact that this protocol limited TRCF treatment to the NV only, and that participants sought supplemental ENT procedures including inferior turbinate and septal swell body reduction suggests that a more comprehensive TCRF treatment protocol including additional target areas such as the inferior turbinate and septal swell body could be beneficial compared to one that is limited to the NV. Notably, the device has already been shown to be effective for the treatment of the septal swell body.^11^ Interestingly, in this study, significantly more patients who were included in the 36‐month follow‐up study had septal swell body hypertrophy and inferior turbinated hypertrophy compared to the nonparticipant cohort, although only the NV was treated per the protocol. Further studies evaluating TCRF in a comprehensive NAO treatment protocol addressing different contributing factors to NAO and across a broader range of NAO symptom severity would help further understand the full potential of TCRF‐based treatments of NAO, to determine an optimal treatment algorithm.

Although the safety and efficacy of TCRF treatment of the NV on NAO symptoms is established,^5^, ^6^ there is still a need for long‐term follow‐up data to contribute to the growing body of evidence across different studies to enable future studies of TCRF with other interventions and to provide evidence on long‐term safety.

Despite the strengths of this study, there are limitations. This was an open‐label, single‐arm, multicenter study, which could result in personnel bias/differences among the study team and/or the physician. Although outcomes were self‐reported at all sites, variability was limited by using a standardized treatment protocol and physician training. Therefore, all participants underwent the same treatment across all sites, and all outcomes were self‐reported by the participants. The absence of in‐person nasal examinations beyond the 3‐month follow‐up is another limitation of the safety portion of this study. Although no device‐ or procedure‐related AEs were reported between the 24‐ and 36‐month follow‐up periods, it is important to note that complications such as intranasal adhesions cannot be definitively ruled out based solely on patient‐reported outcomes. Future studies with standardized in‐person nasal examinations during long‐term follow‐up could provide more robust safety data.

Another limitation is that medication use was not restricted for the participants in this study due to its pragmatic study design. A total of 8 (12.1%) of participants started new medication after baseline, including antihistamines (4.5%), nasal steroid sprays (4.5%), nasal anticholinergic sprays (1.5%), and “other” (1.5%). Notably, all had rhinitis which can contribute to NAO but considered different as the patients in the current study received treatment in the lateral wall in the NV region (Supplemental Table S6, available online). The study was designed to evaluate the effectiveness of TCRF for treating NAO, specifically the NV, not rhinitis, which may have been better addressed by treating additional anatomic structures, such as the inferior turbinate. This limitation is similar to the case of participants who underwent additional ENT procedures after the study procedure, suggesting that targeting other anatomic contributors with TCRF may provide additional benefits. A future registry study could provide real‐world evidence by examining outcomes where multiple anatomic locations are treated in combination.

Interestingly, despite these limitations, many participants reduced their medication use over time (Table 2). Compared to baseline, at 36 months, 34.6% of participants had either stopped or reduced their dose of antihistamine, 50% of participants had either stopped or reduced their dose of decongestant, 58.3% of participants had either stopped or reduced their dose of leukotriene inhibitors, 57.7% of participants had either stopped or reduced their dose of nasal steroid nasal sprays, and 66.6% of participants had stopped their dose of other medication. However, eight patients started new medication after baseline.

Although the use of medications was not controlled, the result of this study adds to the body of knowledge that medications do not confound the treatment effect delivered by the TCRF device. It is also notable that the 2023 Position Statement of the American Academy of Otolaryngology–Head and Neck Surgery^13^ points to NV repair rather than medication use as a means for addressing NV dysfunction.

Long‐term follow‐up studies, such as this one, often face the challenge of high attrition rates, which represent a significant limitation. In this study, participants were excluded due to factors such as undergoing additional procedures or not having severe disease at baseline. These exclusions contributed to a higher treatment response rate. When these participants were included in the sensitivity analysis as nonresponders, the response rate decreased to 73.9% compared to 83.3% based on the primary study endpoint. Nevertheless, long‐term studies such as this study are valuable for identifying possible late side effects of the procedure. In addition, long‐term outcome studies can enable long‐term cost‐benefit estimates, provide information for policy decisions, and allow evaluation of long‐term health effects.

Despite the high attrition rate in our study, our analysis shows that there was no significant difference in NOSE score between patients enrolled for the 36‐month follow‐up (participants) and those who did not (nonparticipants) at their last reported follow‐up visit, yet, there remains a possibility that those who did not enroll may have had a recurrence of nasal obstruction symptoms and it is unknown if they required further treatment. Finally, the study did not include objective measures to evaluate the impact of TCRF on NAO. In this study, more than 50% of the participants had NV narrowing at baseline. TCRF treats NVC through remodeling of the soft tissue during treatment, and contraction occurs during the healing process. In addition to stabilizing the NV, this process can result in a more open NV angle and lead to less resistance to airflow from NV tissue. Future studies incorporating an objective measure, such as rhinometry measurements, 3D computational models, or an active comparator, may provide additional information with which to evaluate the potential benefits of TCRF treatment of the NV in the context of existing treatment options.

## Conclusion

TCRF treatment of the NV resulted in a sustained improvement in NAO symptoms at 36 months following a single procedure with no late device‐ or procedure‐related AEs and reduced medication use at 36‐month postprocedure. This study's results confirm the TCRF procedure's long‐lasting effect on participants with NAO attributed to NVC.

## Author Contributions

**William C. Yao**, study design, conduct, analysis, writing, and manuscript review; **Randall Ow**, study design, conduct, analysis, writing, and manuscript review; **Michael J. Sillers**, study conduct, analysis, and critical manuscript review; **Nathan E. Nachlas**, study conduct, analysis, and critical manuscript review; **Curtis D. Johnson**, study conduct, analysis, and critical manuscript review; **Dale Ehmer**, study conduct, analysis, and critical manuscript review; **Jordan Pritikin**, study conduct, analysis, and critical manuscript review; **Henry P. Barham**, study conduct, analysis, and critical manuscript review.

## Disclosures

### Competing interests

William C. Yao: consultant to Aerin Medical, Acclarent Inc, speaker for Optinose; Randall Ow: consultant to Aerin Medical, Lyra Therapeutics, Optinose, Medical Metrics and Sanofi‐Regeneron, speaker's bureau for GSK, Sanofi‐Regeneron and Optinose; Michael J. Sillers: consultant to Aerin Medical and Neurent Medical; Nathan E. Nachlas: no disclosures reported; Curtis D. Johnson: consultant to Aerin Medical; Dale Ehmer: no disclosures reported; Jordan Pritikin: consultant to Aerin Medical, 3‐D Matrix, Olympus North America, AIM Specialty Health, speaker for Optinose; Henry P. Barham: consultant to Aerin Medical.

### Funding source

The study was funded by Aerin Medical.

## Supporting information


Supporting Information

## Data Availability

Data will not be made available in a public repository but may be made available upon request.
