# Supplementary material for: Three‐Year Outcomes After Temperature‐Controlled Radiofrequency Treatment of Nasal Airway Obstruction
Source: OTO Open. 2025 Apr 7;9(2):e70111. doi: 10.1002/oto2.70111 (PMC11973577; doi:10.1002/oto2.70111)
Supplement: Supplementary file 1 — Supporting Information [file OTO2-9-e70111-s001.docx]

**Supplemental Material**

S**upplemental Table 1. Study eligibility criteria**

| **Inclusion criteria** | **Exclusion criteria** |
| --- | --- |
| 1. Age 18 or older. 2. Willing and able to provide informed consent. 3. Willing and able to comply with the study protocol. 4. Seeking treatment for nasal obstruction. 5. NOSE score of ≥60 at baseline. 6. Nasal valve is a primary or significant contributor to the subject’s nasal obstruction as determined by the study investigator (based on clinical presentation, physical examination, nasal endoscopy, etc.) and the subject has a positive response to any of the following temporary measures (based on patient history or office exam): 7. Use of external nasal dilator strips (e.g., Breathe Right Strips). 8. Q-Tip test (manual intranasal lateralization). 9. Use of nasal stents. 10. Cottle Maneuver (manual lateral retraction of the cheek). | 1. Prior surgical treatment of the nasal valve. 2. Rhinoplasty, septoplasty, inferior turbinate reduction or other surgical nasal procedures within 3 months prior. 3. Anatomy that required an adjunctive surgical nasal procedure on the same day or 3 months after the study procedure. 4. Medical conditions which, in the opinion of the treating physician, would predispose the patient to poor wound healing or increased surgical risk. |

**Supplemental Table 2. Reported adverse events characterized as possibly related to the device and/or procedure**

| **Adverse event** | **Severity** | **Related**  **to study device** | **Related to study procedure** | **Day of event onset^a^** | **Days to resolved/recovered** |
| --- | --- | --- | --- | --- | --- |
| Nasal crusting | Mild | No | Possible | 2 | 12 |
| Nasal crusting | Severe | Probably | Probably | 0 | 144 |
| Nasal crusting | Moderate | Probably | Possible | 0 | 14 |
| Nasal crusting | Moderate | No | Possible | 8 | 14 |
| Nasal soreness/pain | Moderate | No | Possibly | 8 | 14 |
| Nasal congestion/obstruction | Moderate | No | Possibly | 0 | 97 |
| Nasal Injury | Moderate | Probably | Probably | 7 | N/a^b^ |
| Internal nasal swelling/edema | Moderate | No | Possible | 1 | 5 |
| Anosmia | Moderate | No | Possibly | 8 | 14 |
| Sinusitis | Moderate | No | Possible | 88 | 21 |
| Sinusitis | Moderate | Possibly | Possibly | 0 | 42 |
| Vasovagal reaction | Mild | No | Definitely | 0 | 0 |

^a^ Number of days from procedure to AE onset

^b^ Ongoing at time of study exit

Abbreviations: N/a= Non-applicable

**Supplemental Table 3. Reasons patients declined to enroll in the extended 36-month follow-up study**

| **Reasons for not participating** | **No of patients** |
| --- | --- |
| Declined extended follow-up with no reason provided | 7 |
| Busy schedule | 5 |
| Did not return consent form / respond follow-up request | 2 |
| Wanted to participate in another study | 1 |
| Felt they couldn’t provide any further information | 1 |
| **Total** | **16** |

**Supplemental Table 4.** **Details for patients with additional nasal procedures**

|  |  |  |  |  |  | **NOSE Scale Score** | | | | |
| --- | --- | --- | --- | --- | --- | --- | --- | --- | --- | --- |
| **Nasal valve collapse** | **Concomitant conditions** | **Prior nasal surgery** | **Additional procedure after TCRF treatment** | **Reason for additional procedure^a^** | **Days after TCRF treatment** | **Baseline** | **Prior to additional procedure** | **Responder prior to additional procedure^b^** | **24 Mo** | **36 Mo** |
| **Patients participating in the study through 36 Months** | | | | | | | | | | |
| Bilateral dynamic | Bilateral ITH, Left SSB | None | FESS, bilateral turbinate reduction | Frequent sinus infections, progressing nasal disease | 624 | 100 | 30 | Yes | 0 | 10 |
| Bilateral  dynamic | None | Bilateral superior turbinate reduction, middle turbinate reduction, septoplasty | Bilateral inferior turbinate reduction | Worsening nasal airway obstruction | 455 | 80 | 70 | Yes | 55 | 100 |
| Bilateral dynamic | None | None | Bilateral SSB ablation | Worsening nasal airway obstruction | 112 | 80 | 35 | Yes | 15 | 10 |
| Bilateral  static | None | None | Bilateral nasal valve repair | Inadequate relief from study treatment | 567 | 70 | 40 | Yes | 20 | 0 |
| Bilateral  static | Bilateral nasal vestibular stenosis | Sinuplasty | Bilateral PNN TCRF neurolysis | Worsening nasal airway obstruction | 273 | 75 | 40 | Yes | 60 | 50 |
| Unilateral  static | None | Sinuplasty, septoplasty, inferior turbinate reduction | Bilateral PNN TCRF neurolysis | Worsening nasal airway obstruction | 441 | 85 | 60 | Yes | 30 | 35 |
| Bilateral  static | Bilateral nasal vestibular stenosis | None | Septoplasty, bilateral inferior turbinate reduction | Worsening nasal airway obstruction | 241 | 65 | 65 | No | 40 | 25 |
| Bilateral dynamic | Bilateral nasal vestibular stenosis | PNN cryoablation, inferior turbinate reduction | Bilateral maxillary balloon sinuplasty, septoplasty with spreader graft to treat nasal vestibular stenosis, swell body reduction | Worsening nasal airway obstruction, inadequate relief from study procedure | 373 | 90 | 95 | No | 0 | 0 |
| Bilateral dynamic | Bilateral nasal vestibular stenosis | None | Septoplasty and nasal valve surgery | Inadequate Relief from Study Procedure | 217 | 95 | 95 | No | 55 | 15 |
| **Patients participating in the study through 24 Months** | | | | | | | | | | |
|  |  |  |  |  |  | **NOSE Scale Score** | | | | |
| **Nasal valve collapse** | **Concomitant conditions** | **Prior nasal surgery** | **Additional procedure after TCRF treatment** | **Reason for additional procedure^a^** | **Days after TCRF treatment** | **Baseline** | **Prior to additional procedure** | **Responder prior to additional procedure^b^** | **24 Mo** | **36 Mo** |
| Bilateral  static | Bilateral nasal vestibular stenosis, right septal deviation | Bilateral septoplasty, bilateral inferior turbinate reduction, bilateral sinuplasty | Septoplasty | Worsening nasal airway obstruction | 104 | 80 | UNK^d^ | UNK^d^ | UNK^c^ | UNK^c^ |
| Bilateral dynamic | Bilateral nasal vestibular stenosis, bilateral septal deviation | None | Septoplasty, nasal valve repair (bioabsorbable implant), outfracture turbinate | Worsening nasal airway obstruction | 360 | 75 | 45 | Yes | UNK^e^ | UNK^e^ |
| Bilateral  static | Bilateral nasal vestibular stenosis | Bilateral septoplasty, bilateral sinuplasty, bilateral inferior turbinate reduction | Septoplasty | Worsening nasal airway obstruction | 101 | 85 | 60 | Yes | 70 | DCL |
| Bilateral dynamic | Bilateral nasal vestibular stenosis | Right partial ethmoidectomy, right antrostomy/tissue removal | Left nasal valve repair (alar batten graft with auricular cartilage) | Inadequate relief from study procedure | 350 | 95 | 90 | No | UNK^e^ | UNK^e^ |
| Bilateral dynamic | Bilateral nasal vestibular stenosis | Bilateral septoplasty, bilateral polyp removal | Inferior turbinate reduction | Worsening nasal airway obstruction | 292 | 85 | 80 | No | 0 (at 12 Mo) | UNK^c^ |

^a^ Noted in study case report form

^b^ Responder per the study definition prior to the additional procedure

^c^ Lost to follow-up, was a responder at 6 months (NOSE Scale score improvement of 20% or 1 severity category improvement)

^d^ Missed 3-month visit

^e^ Withdrew

Abbreviations: DCL=declined participation, FESS = functional endoscopic sinus surgery, ITH = inferior turbinate hypertrophy, MO-month, PNN = posterior nasal nerve, SSB = septal swell body, TCRF = temperature-controlled radiofrequency, UNK = unknown

**Supplemental Table 5. Exploratory analysis on severity distribution across study timepoints**

| **Comparison** | **Mean estimate** | **Mean confidence limits** | **Chi-square** | **P-value** |
| --- | --- | --- | --- | --- |
| 3M vs BL | 0.027 | 0.0133 | 94.78 | <.0001 |
| 6M vs BL | 0.0274 | 0.0133 | 89.81 | <.0001 |
| 12M vs BL | 0.0226 | 0.0105 | 89.7 | <.0001 |
| 24M vs BL | 0.0273 | 0.0127 | 80.19 | <.0001 |
| 36M vs BL | 0.034 | 0.016 | 72.37 | <.0001 |
| 3M vs 6M | 0.5041 | 0.4086 | 0.01 | 0.9329 |
| 3M vs 12M | 0.4544 | 0.3511 | 0.69 | 0.4064 |
| 3M vs 24M | 0.5035 | 0.3945 | 0 | 0.9504 |
| 3M vs 36M | 0.5594 | 0.4403 | 0.96 | 0.3282 |
| 6M vs 12M | 0.4503 | 0.3709 | 1.41 | 0.2349 |
| 6M vs 24M | 0.4994 | 0.401 | 0 | 0.99 |
| 6M vs 36M | 0.5553 | 0.4609 | 1.32 | 0.2505 |
| 12M vs 24M | 0.549 | 0.4595 | 1.15 | 0.2831 |
| 12M vs 36M | 0.6038 | 0.5111 | 4.8 | 0.0285 |
| 24M vs 36M | 0.556 | 0.452 | 1.11 | 0.2911 |

BL = baseline; M= month

**Supplemental Table 6.** **Exploratory sensitivity analyses using non-responder imputation in participants and non-participants***

|  | 1. ***Treatment Responders* (per-protocol eligible - 36-month cohort - includes those with another ENT procedure imputed as nonresponders)*** | | | | | | | | | | | | | | | | | |
| --- | --- | --- | --- | --- | --- | --- | --- | --- | --- | --- | --- | --- | --- | --- | --- | --- | --- | --- |
| **Treatment Responder >= 20% or 1+ categories** | **3 Months** | | | | **6 Months** | | | | **12 months** | | | | **24 months** | | | | **36 months** | |
|  | **36-month participants (imputed)** | | **nonparticipant** | | **36-month participants (imputed)** | | **nonparticipant** | | **36-month participants (imputed)** | | **nonparticipant** | | **36-month participants (imputed)** | | **nonparticipant** | | **36-month participants (imputed)** | |
|  | **N** | **%** | **N** | **%** | **N** | **%** | **N** | **%** | **N** | **%** | **N** | **%** | **N** | **%** | **N** | **%** | **N** | **%** |
| **Yes** | 70 | 94.6 | 39 | 86.7 | 66 | 88.0 | 32 | 72.7 | 65 | 86.7 | 24 | 72.7 | 59 | 78.7 | 22 | 88.0 | 55 | 73.3 |
| **No** | 4 | 5.4 | 6 | 13.3 | 9 | 12.0 | 12 | 27.3 | 10 | 13.3 | 9 | 27.3 | 16 | 21.3 | 3 | 12.0 | 20 | 26.7 |
| **n evaluated** | 74 | 100.0 | 45 | 100.0 | 75 | 100.0 | 44 | 100.0 | 75 | 100.0 | 33 | 100.0 | 75 | 100.0 | 25 | 100.0 | 75 | 100.0 |
| **Treatment Responder >= 20% or 1+ categories** | 1. ***Treatment Responders* (per-protocol eligible - 36-month cohort - includes those with another ENT procedure and those severe/extreme condition imputed as nonresponders)*** | | | | | | | | | | | | | | | | | |
|  | **3 Months** | | | | **6 Months** | | | | **12 months** | | | | **24 months** | | | | **36 months** | |
|  | **36-month participants (imputed)** | | **nonparticipant** | | **36-month participants (imputed)** | | **nonparticipant** | | **36-month participants (imputed)** | | **nonparticipant** | | **36-month participants (imputed)** | | **nonparticipant** | | **36-month participants (imputed)** | |
|  | **N** | **%** | **N** | **%** | **N** | **%** | **N** | **%** | **N** | **%** | **N** | **%** | **N** | **%** | **N** | **%** | **N** | **%** |
| **Yes** | 62 | 83.8 | 35 | 77.8 | 59 | 78.7 | 28 | 63.6 | 58 | 77.3 | 22 | 66.7 | 51 | 68.0 | 19 | 76.0 | 49 | 65.3 |
| **No** | 12 | 16.2 | 10 | 22.2 | 16 | 21.3 | 16 | 36.4 | 17 | 22.7 | 11 | 33.3 | 24 | 32.0 | 6 | 24.0 | 26 | 34.7 |
| **n evaluated** | 74 | 100.0 | 45 | 100.0 | 75 | 100.0 | 44 | 100.0 | 75 | 100.0 | 33 | 100.0 | 75 | 100.0 | 25 | 100.0 | 75 | 100.0 |

***Legend:** Any patient who made a visit and had a NOSE score after an additional ENT procedure was considered a nonresponder, regardless of the score. A total of 14 patients underwent additional ENT procedures; of these, 12 patients had NOSE scores after the additional ENT procedure. Among these, 9 patients were included in the 36-month cohort with a 36-month NOSE score available, while 3 patients did not have a NOSE scores available after the additional ENT procedure. Two patients were withdrawn after the additional ENT procedure and did not have any subsequent visit scores to impute as nonresponders; therefore, they did not qualify for the 36-month cohort. Patients lost to follow-up (LTF) with no subsequent visit were not imputed as nonresponders.

**Supplemental Table 7. Patient characteristics and NOSE scale scores for study participants initiating medication post-procedure**

|  |  |  |  |  | **NOSE Scale Score** | | | | |
| --- | --- | --- | --- | --- | --- | --- | --- | --- | --- |
| **Comorbid conditions reported at baseline** | **Medication** | **When medication was reported as started** | **Indication** | **TCRF**  **Procedure** | **Baseline** | **Prior to additional medications** | **Responder prior to additional medications^a^** | **24 Mo** | **36 Mo** |
| Allergies | Anticholinergic | 19-Jun 2023 (Unscheduled) | Postnasal drip | 5/9/2020 | 80 | 100 | No | 100 | 100 |
| Allergic rhinitis | Antihistamine | 22-Sep 2020  (3M FU visit) | Allergic rhinitis | 6/5/2020 | 80 | 50 | Yes | 0 | 60 |
| N/A | Antihistamine | Feb-2022  (24M FU visit) | Allergies | 8/5/2020 | 100 | 85 | No | 85 | 0 |
| Seasonal allergies | Antihistamine | Jan-2021  (12M FU visit) | Allergic rhinitis | 5/6/2020 | 65 | 15 | Yes | 5 | 10 |
| Allergies | Steroid spray | 15-Jul 2021  (36M FU visit) | Allergies | 6/26/2020 | 90 | 10 | Yes | 25 | 10 |
| N/A | Steroid spray | Unknown-2022  (24M FU visit) | Allergies | 7/23/2020 | 85 | 50 | Yes | 45 | 40 |
| N/A | Steroid spray | 17-Sep 2020  (6M FU visit) | Rhinitis | 3/16/2020 | 100 | 65 | Yes | 55 | 45 |
| N/A | Oral combination | 20-Sep 2020  (6M FU visit) | Allergic rhinitis | 3/30/2020 | 85 | 40 | Yes | 0 | 0 |

^a^ Met criteria for treatment responder per protocol treatment r prior to the additional medication

Abbreviations: FU=follow-up, TCRF=temperature-controlled radiofrequency, N/A= non-applicable
